# Impact of Folic Acid in Modulating Antioxidant Activity, Osmoprotectants, Anatomical Responses, and Photosynthetic Efficiency of *Plectranthus amboinicus* Under Salinity Conditions

**DOI:** 10.3389/fpls.2022.887091

**Published:** 2022-07-22

**Authors:** Omar A. A. I. Al-Elwany, Khaulood A. Hemida, Mohamed A. Abdel-Razek, Taia A. Abd El-Mageed, Mohamed T. El-Saadony, Synan F. AbuQamar, Khaled A. El-Tarabily, Ragab S. Taha

**Affiliations:** ^1^Horticulture Department, Faculty of Agriculture, Fayoum University, Fayoum, Egypt; ^2^Botany Department, Faculty of Science, Fayoum University, Fayoum, Egypt; ^3^Soil and Water Department, Faculty of Agriculture, Fayoum University, Fayoum, Egypt; ^4^Department of Agricultural Microbiology, Faculty of Agriculture, Zagazig University, Zagazig, Egypt; ^5^Department of Biology, College of Science, United Arab Emirates University, Al-Ain, United Arab Emirates; ^6^Khalifa Center for Genetic Engineering and Biotechnology, United Arab Emirates University, Al-Ain, United Arab Emirates; ^7^Harry Butler Institute, Murdoch University, Murdoch, WA, Australia; ^8^Botany Department, Faculty of Agriculture, Beni-Suef University, Beni-Suef, Egypt

**Keywords:** folic acid, leaf osmoprotectant growth, oil yield, photosynthetic productivity, saline water

## Abstract

Salinity is a major threat to the sustainability of agricultural production systems. Salt stress has unfavorable implications on various plant physio-morphological and biochemical reactions, causing osmotic and ionic stress. Exogenously applied folic acid (FA) may at least provide one mechanism to evade the injurious stress effects of saline irrigation water on *Plectranthus amboinicus*. In this regard, two pot trials were performed during the 2018–2019 and 2019–2020 seasons in an open greenhouse of an experimental farm (29°17'N; 30°53'E) in Fayoum, Egypt. We tested four levels of saline irrigation water (SW): 34, 68, and 102 mM NaCl, plus tap water as the control = 0), combined with FA at three concentrations (25 and 50 μM, plus spray with distilled water as the control = 0). The growth parameters, biochemistry, physiology, elemental leaf status, essential oil content, and anatomical responses were assessed. Salt markedly reduced photosynthetic productivity [Fv/Fm and performance index (PI)], total chlorophyll [soil plant analysis development (SPAD)], and leaf osmoprotectant compounds, i.e., total soluble sugars (TSS), free amino acids, proline, and total phenolics, thus hampering *P. amboinicus* growth and essential oil yield. However, the addition of FA as a foliar spray to *P. amboinicus* irrigated with saline water induced increases in Fv/Fm, SPAD, and PI. These were linked with enriched stem anatomical structures, leaf osmoprotectant compounds, and enhanced leaf enzymatic activity, e.g., superoxide dismutase, catalase, ascorbate peroxidase, glutathione reductase, glutathione, ascorbic acid, and antioxidant content. Under salt stress, supplementation of 25 and 50 μM FA increased the growth and production of essential oil by 27.8 and 55.6%, respectively, compared with no applied FA. The highest growth characteristics and elemental leaf contents were obtained when *P. amboinicus* was irrigated with 0 mM saline water and treated foliarly with 50 μM of FA compared with non-treated plants. Overall, these data showed that foliar spraying with FA reduces the impact of salt stress on *P. amboinicus* irrigated with saline water.

## Introduction

*Plectranthus* is a large genus of the family Lamiaceae, including around 300 species of *Plectranthus* native to tropical Africa, Asia, and Australia (Lukhoba et al., 2006). Globally, *Plectranthus* spp. are well known and are extensively used in traditional medicine. Many species of *Plectranthus* are used to treat digestive system disturbance, including vomiting, diarrhea, mouth and throat infections, stomach pain, and nausea (Lukhoba et al., 2006). Some species are also employed as purgatives, carminatives, and anthelmintic remedies (Githinji and Kokwaro, 1993). *P. amboinicus* is the most commonly-used species for treating burns, injuries, sores, insect bites, and sensitivities (Chifundera, 2001). Common names of *P. amboinicus* in English include Indian borage, country borage, French thyme, Indian mint, Mexican mint, Cuban oregano, soup mint, Spanish thyme (Bañuelos-Hernández et al., 2020). The leaf extract is used to make a forskolin-like chemical utilized in hair coloring (Kanne et al., 2015). Furthermore, it yields an essential oil with anti-allergenic properties *via* passive cutaneous anaphylaxis suppression (Bañuelos-Hernández et al., 2020). *Plectranthus* spp. are reported to have cytotoxic and anti-tumor properties and can be used to treat cancer (Lukhoba et al., 2006).

Salinity is distinctive environmental stress faced by plants in arid and semi-arid environments. Yield restriction resulting from salinity in soil or irrigation water has led to the desertification of about one-third of the irrigated land worldwide (Shaaban et al., 2022). In response to salt stress, morphological and biochemical changes are imposed (Ali et al., 2013) due to a reduction in the water supply to leaf tissues (Abd El-Mageed et al., 2019). Consequently, plant root length and mass are reduced (Shannon and Grieve, 1998). A reduction in leaf area could be ascribed to a decrease in turgor due to changes in cell elongation and division or a reduction in photosynthetic degree (Abd El-Mageed et al., 2019). In salt-stressed plants, reactive oxygen species (ROS), such as superoxide (${\text{O}}_{2}^{•-}$), hydrogen peroxide (H_2_O_2_), and hydroxyl (OH^•^) radicals, are overproduced from chloroplasts during photosynthesis (Abdou et al., 2021), leading to lipid and protein destruction and dysfunctional DNA (Yasar et al., 2006). As well, chlorophyll degradation and membrane lipid peroxidation occurs (Yildirim et al., 2008). The rapid removal of the harmful impacts of ROSs is an effective stress-defense mechanism. Antioxidant defense systems play an important role in this regard (Abd El-Mageed et al., 2018).

Salinity causes both osmotic and oxidative stress in higher plants; therefore, they have evolved complicated coping systems. These mechanisms are accomplished by either external uptake of organic compounds or by synthesizing favorable solutes, such as amino acids, sugars, and vitamins that act as osmolytes (Rady et al., 2021a). These activate protein configuration and membrane stabilization against denaturation (Munns, 2002); furthermore, they prevent water loss and maintain cell turgor under salt stress (Rady et al., 2021b). The oxidative stress mechanism occurs when ROS production represses the antioxidant system capacity (Semida et al., 2021). For these reasons, plants evolved multi-antioxidant defense mechanisms, including the antioxidants glutathione, ascorbate, β-carotene, and α-tocopherol; besides, enzymes like superoxide dismutase (SOD), catalase (CAT), ascorbate peroxidase (APX), phenol peroxidase (GPX), and glutathione reductase (GR) substitute as ROS scavengers (Semida et al., 2015b).

Folic acid (FA, folates), also called vitamin B9, is an important constituent of metabolism in all living organisms (Bekaert et al., 2008) and is a donor/acceptor for one-carbon transfer reactions taking place in the formation of many significant biomolecules, such as amino acid metabolism and nucleic acid synthesis (Gorelova et al., 2017), which enables plants to synthesize proteins (Poudineh et al., 2015). In the methyl cycle, reactions catalyzed by FA through providing methyl groups are indispensable in regulating gene expression but are also prerequisites for the synthesis of lipids, proteins, chlorophyll, and lignin (Gorelova et al., 2017).

During the past decades, *P. amboinicus* has been subjected to many agronomical and environmental studies. However, no available studies have evaluated the role of FA in the adaptation of plants under saline water (SW) irrigation.

Consequently, the current study aimed to determine the strategies through which *P. amboinicus* seedlings withstand salinity. In this investigation, the growth attributes, leaf osmoprotectant compounds, essential oil yield, leaf enzymatic activity and antioxidants, and stem and leaf anatomical structures of *P. amboinicus* plants increased when FA was imposed under SW irrigation. This was assessed by investigating the physio-morphological changes induced by salinity stress, and using FA to enhance essential oil yield and salt tolerance in *P. amboinicus* seedlings exposed to different saline irrigation water levels.

## Materials and Methods

### Trial Site, Climatic Conditions, and Growing Media

Two pot experiments were performed in an open greenhouse of the experimental farm (southeast Fayoum; 29°17'N; 30°53'E), of the Faculty of Agriculture, Fayoum University, Fayoum, Egypt, throughout the seasons of 2018 and 2019. The average climatic conditions throughout the two experimental periods (March 6–May 26) were the average one-day and night temperatures of 32 ± 3°C and 18 ± 2°C, respectively. Relative humidity was 65 ± 4%, and daylight interval averaged 13 h. Natural sunlight conditions were suitable throughout the experiment for all plant growth periods. Pots (40 cm in diameter and 35 cm deep) were filled with homogenized peat moss, vermiculite, and sand (2:1:1 v/v) as substrates for the growing media.

### Plant Material, Trial Design, and Cultural Practices

*P. amboinicus* cuttings were rooted on a plastic box (10 cm in diameter) in the growth chamber next to the greenhouse in February 2018 and 2019. After 2 weeks, when seedlings had an intact and healthy root, they were resettled into black plastic pots on 6 March of the two respective seasons. *P. amboinicus* seedlings were allowed to establish for 21 days after transplantation (DAT) before irrigation with SW on 27 March. During this period, the developed shoots were pinched over the 3^rd^ leaf for branching. Before transplantation, all pots of all treatments were set in a completely randomized form in a factorial scheme in an open greenhouse.

Regardless of the irrigation with SW levels as an experimental factor, standard agronomic practices were performed according to need, including fertilization and pest and disease control. Before transplantation, the growing media in each pot (1.4 L) was supplemented with 1.5 g of Nutri-Leaf NPK fertilizer (20, 20, and 20% of N, P_2_O_5_, and K_2_O, respectively) (Agro-Consult Co., Corniche El-Neil, Cairo, Egypt). After transplantation, the fertilizer was regularly applied once a week for all plants of all treatments as a foliar spray of 1 g l^−1^ of Kristalon (20, 5, 10, and 2% of N, P_2_O_5_, K_2_O, and Mg, respectively) (YARA Agri, Staré Město, Czech Republic) starting from 20 DAT and through the end of the flowering stage.

### Treatments and Other Practices

For both seasons, we started saline irrigation treatments [SW: 34, 68, and 102 mM NaCl, and control (0; tap water)] after 21 days of transplantation (27 March) to the end of the experiment (May 26). The *P. amboinicus* plants were irrigated twice a week. Seedlings of each salinized-water stress level were regularly sprayed with foliar FA at concentrations of 0, 25, and 50 μM (0 = control = spray with distilled water). Sixty pots were allocated for each SW treatment. The pots for each water treatment were divided into three groups, each with 20 pots occupied by three levels of FA. Therefore, 12 treatments (4 SW × 3 FA) were applied with three replicates under greenhouse conditions. FA as the foliar spray was applied three times during the experiment (21, 28, and 35 DAT, respectively).

### Plant Sampling

At 45 DAT, five plants were randomly selected from every treatment and cut to ground level for anatomical study. For the agronomical study, at 90 DAT, five *P. amboinicus* plants and their whole root systems were carefully removed from each experimental plot. In addition, five plants from each treatment were chosen at random for dry matter and physio-biochemical evaluations.

### Growth Characteristics

Plants were immersed in a bucket of water and shaken gently to eliminate any adhering growing media, and the length of roots and shoots (cm) were assessed by a meter scale. The number of shoots and leaves on each plant were counted. The stem diameter (mm) was assessed by a Sealy So707-Digital Electronic Vernier Caliper (0–150 mm/0–6″) above the soil surface by 5 cm. The shoot/root systems were weighed to determine their fresh weight (g plant^−1^), then placed in an over at 80°C for 72 h. The dry shoot and dry root weights were recorded (g plant^−1^).

### Determination of Total Chlorophyll, Chlorophyll Fluorescence, and PI of Photosynthesis

Total chlorophyll [soil plant analysis development (SPAD)] was assessed using a Konica Minolta chlorophyll meter [SPAD 502 model (Konica Minolta Optics, Osaka, Japan)] on the third developed leaf from the lateral shoot. Chlorophyll fluorescence (F_v_/F_m_) and the performance index (PI) were assessed on two different sunny days by a portable fluorometer (Handy PEA, Hansatech Instruments Ltd., Kings Lynn, United Kingdom) as previously reported (Clark et al., 2000; Maxwell and Johnson, 2000).

### Determination of Leaf Osmoprotectant Compounds

Total soluble sugars (TSS) were extracted and measured at 625 nm by a Bausch and Lomb-2000 Spectronic Spectrophotometer (Bausch and Lomb analytical systems divisions, Rochester, New York, USA) (Irigoyen et al., 1992). The colorimetric approach described by Bates et al. (1973) was used to identify proline concentration (mg 100 g DW^−1^). Using the methanolic extract of the same leaf material, the total phenolics (mg g DW^−1^) were assessed by the Folin–Ciocalteu colorimetric technique reported by Singleton and Rossi (1965). We used the formula [phenol content (mg catechin g^−1^ DW) = (A_s_/A_c_) × R × 250] of Hashemi et al. (2011) to compute the phenolic contents, where A_s_ and A_c_ represent the absorbance readings of the sample and the catechin, respectively. The percentage of extraction yield was denoted by R. Soluble proteins were extracted according to the method described by Hassanein (1977). Then, the extractable was identified according to the method adopted by Bradford (1976). Free amino acids were extracted and determined according to the methods by Yemm et al. (1955) and Vartanian et al. (1992).

### Extraction of Essential Oil

The essential oil was assessed by the hydro-distillation method wherein the aerial parts of dried plants (100 g) in a modified Clevenger apparatus for 3 h of extraction (Esquível et al., 1999). Subsequently, distillation was stopped, so the essential oil percentage was measured using the dry weight of the aerial parts (biomass yield) of *P. amboinicus* plants.

### Determination of the Enzymatic and Non-Enzymatic Antioxidant Compounds

Fresh *P. amboinicus* leaf (1 g) samples were homogenized in liquid N_2_ with 0.05 M phosphate buffer (pH 7.0) containing 0.1 M EDTA and 1% PVP at 4°C by a mortar and pestle, followed by centrifugation at 4°C in a Beckman Coulter refrigerated centrifuge (Brea, California, USA) at 15,000 × g for 15 min (Garratt et al., 2002). Then, the concentration of SOD, APX, CAT, and GR was assessed according to the methods of Beauchamp and Fridovich (1971), Nakano and Asada (1981), Thomas et al. (1982) and Aebi (1984). The performance of non-enzymatic, e.g., ascorbic acid (AsA) and glutathione (GSH), was determined according to Jablonski and Anderson (1978) and Kampfenkel et al. (1995).

### Determination of Mineral Content of Leaves

Using the same leaf material, the N content (%) was assessed (Donald and Robert, 1998). The P content (mg g^−1^ DW) was quantitatively measured using the molybdenum-reduced molybdophosphoric blue color method (Jackson, 1973) in sulfuric acid, and using diluted sulfomolybdic acid and sodium bisulphite-H_2_SO_4_ solutions as reagents. The contents of K^+^ and Na^+^ (mg g^−1^ DW) were measured using a Perkin-Elmer Model 52-A Flame Photometer (PerkinElmer, Inc., Waltham, Massachusetts, USA) (Wilde et al., 1985). The leaf Cl^−^ content (mg g^−1^ DW) was measured using a Perkin-Elmer Atomic Absorption Spectrophotometer (PerkinElmer, Inc.) (Higinbotham et al., 1967).

### Anatomical Study

For the anatomical measurement, leaf and stem samples were taken at 50 DAT. Measurements of stem diameter, cortex thickness, phloem thickness, xylem thickness, vessel diameter, and pith thickness were measured with the AnalySIS® 3.2 software program for image analysis. The fragments were detected and recognized according to Sass (1961) using an upright light microscope (AxioPlan, Zeiss, Jena, Germany).

### Statistical Analysis

All data were displayed as means ± standard errors. Combined analysis for the two study periods was conducted according to the homogeneity of experimental error variance. Duncan's Multiple Range Test was used to identify the significant differences among means, which were compared at *p* ≤0.05 by INFOSTAT computer software (v.2019 statistical package, Córdoba University, Córdoba, Argentina).

## Results

### Growth Parameters

Different sodium chloride (NaCl) concentrations of SW-irrigated *P. amboinicus* plants significantly decreased growth parameters in terms of shoot length (cm), number of shoots plant^−1^, number of leaves plant^−1^, stem diameter (mm), root length (cm), and fresh and dry weight (g) of shoots compared with the unstressed control (tap water; Table 1). It is worth noting that plants irrigated with 102 mM NaCl reflected the lowest shoot length (cm) by 26.9%, number of shoots plant^−1^, by 47.4%, number of leaves plant^−1^, by 51.0%, stem diameter (mm) by 36.2%, root length (cm) by 41.1%, shoot fresh weight (g plant^−1^) by 48.3%, and shoot dry weight (g plant^−1^) by 55.3% compared with those of the unstressed control (SW_0_). About the FA impact, the abovementioned parameters of *P. amboinicus* increased significantly with foliar FA spray at any concentration (i.e., 25 or 50 μM) compared with those of the control (Table 1).

**Table 1:** The effect of SW irrigation and FA spraying on growth characteristics of *Plectranthus amboinicus* plants grown under greenhouse conditions.

**Treatment**	**Shoot length (cm)**	**Number of shoots plant^**−1**^**	**Number of leaves plant^**−1**^**	**Stem diameter (mm)**	**Root length (cm)**	**Shoot weight (g plant** ^**−1**^ **)**	
						**FW**	**DW**
SW (mM)	^*^	^*^	^*^	^*^	^*^	^*^	^*^
0 (Control)	145.8 ± 5.8a	13.3 ± 0.9a	26.5 ± 1.4a	16.3 ± 1.6a	19.7 ± 0.9a	679.3 ± 42.2a	306.4 ± 14.0a
34	135.2 ± 5.5b	11.7 ± 0.6b	21.8 ± 1.1b	14.2 ± 0.9b	16.7 ± 0.9b	526.9 ± 35.0b	260.1 ± 14.7b
68	120.5 ± 5.7c	9.1 ± 0.7c	16.7 ± 0.9c	12.4 ± 1.5c	14.5 ± 0.6c	434.7 ± 35.1c	199.4 ± 15.2c
102	106.5 ± 4.3d	7.0 ± 0.4d	13.0 ± 1.1d	10.4 ± 0.7d	11.6 ± 0.3d	351.3 ± 37.0d	137.1 ± 15.2d
FA (μM)	^*^	^*^	^*^	^*^	^*^	^*^	^*^
0 (Control)	117.6 ± 4.9c	8.1 ± 0.5c	15.0 ± 1.0c	11.6 ± 1.2c	13.5 ± 0.6c	438.7 ± 40.3c	201.0 ± 15.5c
25	127.6 ± 5.0b	10.2 ± 0.6b	19.2 ± 0.7b	13.5 ± 1.4b	15.6 ± 0.4b	503.3 ± 39.1b	226.4 ± 14.5b
50	135.9 ± 6.0a	12.4 ± 0.8a	24.3 ± 1.8a	14.9 ± 1.0a	17.7 ± 1.0a	552.2 ± 32.7a	249.9 ± 14.4a
SW × FA	^*^	^*^	^*^	^*^	^*^	^*^	^*^

* *Indicates significant differences at P ≤ 0.05 probability level*.

*According to the least-significant difference test, means ± SE followed by different letters in each column are significantly different at P ≤ 0.05*.

*SW, saline water; FA, folic acid; FW, fresh weight; DW, dry weight*.

Plants supplemented with FA at 25 or 50 μM increased shoot length (cm) by 8.5 and 15.6%, number of shoots plant^−1^, by 25.9 and 53.1%, number of leaves plant^−1^, by 28.0 and 62.0%, stem diameter (mm) by 16.4 and 28.4%, root length (cm) by 15.6 and 31.1%, shoot fresh weight (g plant^−1^) by 14.7 and 25.9%, and shoot dry weight (g plant^−1^) by 12.6 and 24.3%, respectively, compared with the control. The interaction effect between SW and FA applications was significant for *P. amboinicus* growth parameters (Table 1). In general, the highest growth parameters were obtained from the interactive treatment of non-saline water of SW_0_ (tap water) × FA at 50 μM, followed by irrigation with SW of 34 mM NaCl × FA at 50 μM, then irrigation with SW of 68 mM NaCl × FA at 50 μM, and finally, the lowest irrigation with SW of 102 mM NaCl × FA at 50 μM. Conversely, the lowest growth parameters were obtained from the interactive treatments of the irrigation with SW of 102 mM NaCl × FA at 0.0 μM (distilled water).

### Total Chlorophyll (SPAD), Fv/Fm, and PI

Applied SW at different concentrations (i.e., 34, 68, and 102 mM of NaCl) significantly decreased phytochemical constituents in fresh leaves of *P. amboinicus* plants, including total chlorophyll (SPAD), chlorophyll florescence (Fv/Fm), and PI of photosynthesis compared to the control (tap water) (Table 2). For instance, irrigating plants with SW at 102 mM NaCl decreased SPAD by 28.1%, chlorophyll florescence (Fv/Fm) by 10.5%, and PI of photosynthesis by 54.1% compared with the control (SW_0_). Foliar FA treatment significantly increased the aforementioned phytochemical constituents of *P. amboinicus* using a solution of 25 μM FA by 19.0, 5.1, and 25.4%, respectively. Using a solution of 50 μM FA, the SPAD, Fv/Fm, and PI further increased by 35.8, 6.3, and 56.6%, respectively, compared with the control (Table 2).

**Table 2:** The effect of SW irrigation and FA spraying on SPAD, Fv/Fm, and PI of *Plectranthus amboinicus* plants grown under greenhouse conditions.

**Treatment**	**SPAD**	**Fv/Fm**	**PI**
SW (mM)	^*^	^*^	^*^
0 (Control)	32.99 ± 0.4a	0.86 ± 0.0a	7.30 ± 0.2a
34	29.40 ± 0.3b	0.84 ± 0.0b	6.30 ± 0.4b
68	26.69 ± 0.7c	0.82 ± 0.0c	5.09 ± 0.3c
102	23.71 ± 0.6d	0.77 ± 0.0d	3.35 ± 0.2d
FA (μM)	^*^	^*^	^*^
0 (Control)	23.84 ± 0.4c	0.79 ± 0.0c	4.33 ± 0.3c
25	28.38 ± 0.5b	0.83 ± 0.0b	5.43 ± 0.2b
50	32.38 ± 0.6a	0.84 ± 0.0a	6.78 ± 0.3a
SW × FA	^*^	^*^	^*^

* *Indicates significant differences at P ≤ 0.05 probability level*.

*According to the least-significant difference test, means ± SE followed by different letters in each column are significantly different at P ≤ 0.05*.

*SW, saline water; FA, folic acid; SPAD, total chlorophyll (soil plant analysis development); Fv/Fm, chlorophyll fluorescence; PI, performance index*.

**Figure 1:**
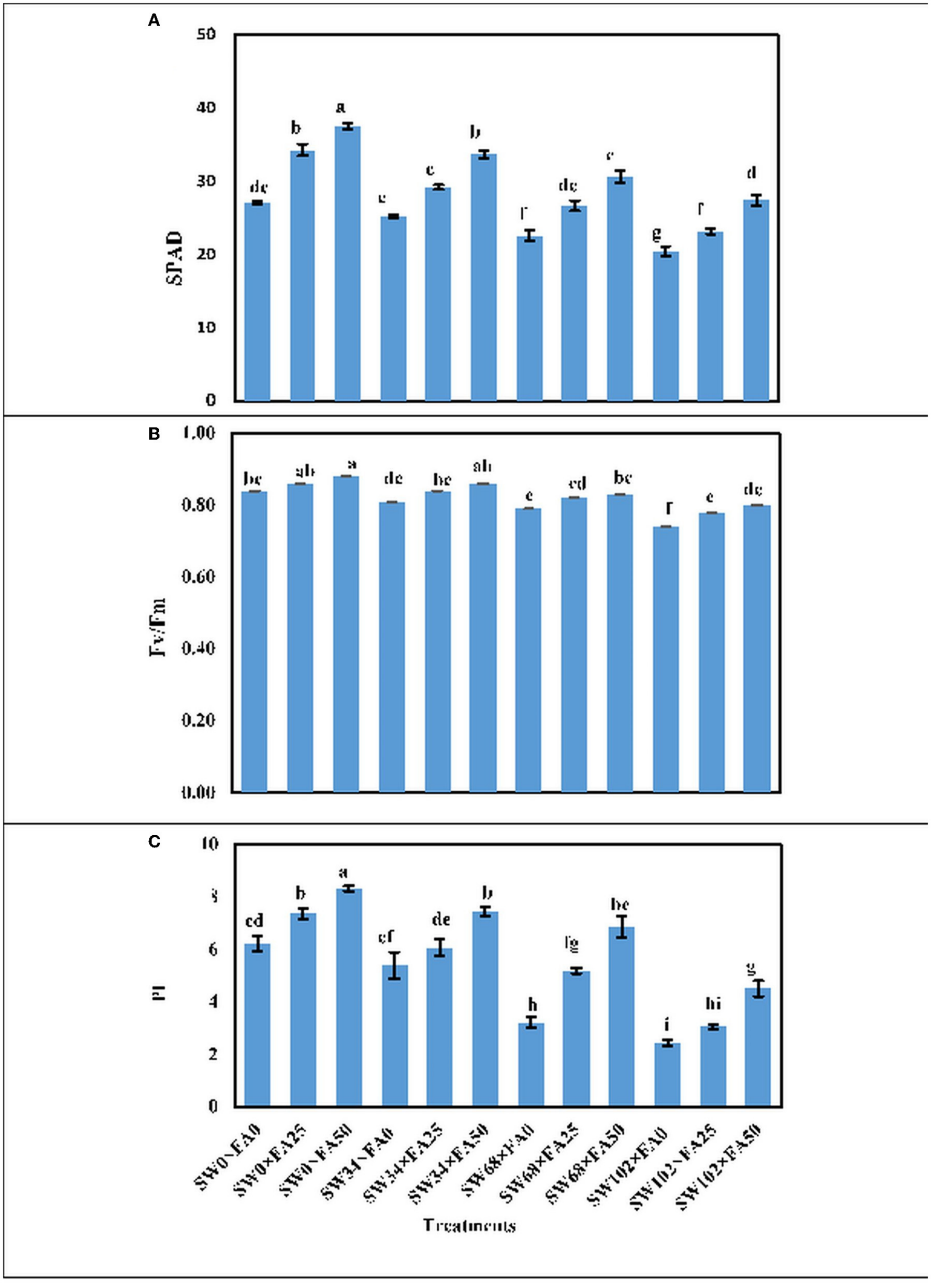
The interactive effect of folic acid (FA) and saline water (SW) on photosynthetic productivity of *Plectranthus amboinicus*. **(A)** SPAD; **(B)** Fv/Fm; and **(C)** PI of *P. amboinicus* measured in the two seasons of 2018/2019 and 2019/2020. Different letters on the bars refers to significant differences among means based on Fisher's least-significant difference test at *p* < 0.05. SPAD, total chlorophyll (soil plant analysis development); *Fv/Fm*, chlorophyll fluorescence; *PI*, performance index; SW0, 0 mM NaCl; SW34, 34 mM NaCl; SW68, 68 mM NaCl; SW102, 102 mM NaCl; FA0, 0 μM FA; FA 25, 25 μM FA; FA50, 50 μM FA.

The interactive effect between SW treatments and FA applications was significant for the phytochemical constituents in fresh leaves of *P. amboinicus* (Figure 1). Generally, the highest phytochemical constituents were recorded from the interactive treatment of non-saline water of SW0 (tap water) × FA at 50 μM, followed by irrigation with SW of 34 mM NaCl × FA at 50 μM, then irrigation with SW of 68 mM NaCl × FA at 50 μM, and finally, the lowest SW irrigation of 102 mM NaCl × FA at 50 μM. The lowest phytochemical constituents were obtained from the interactive treatments of the irrigation with SW of 102 mM NaCl × FA at 0.0 μM (distilled water).

### Leaf Osmoprotectant Compounds and Essential Oil Yield (%)

When the NaCl concentration reached 34 mM, the leaf osmoprotectant compounds of *P. amboinicus* plants, i.e., TSS, free amino acids, proline, and phenolic content (mg g^−1^ DW) increased significantly by 23.8, 29.4, 21.4, and 19.2%, respectively. At 68 mM NaCl, they further increased by 47.6, 70.6, 42.9, and 50.0%, respectively. Conversely, they significantly decreased when the NaCl concentration was elevated to 102 mM, compared with the control (tap water) (Table 3). Protein content (mg g^−1^ DW) and essential oil yield (%) significantly decreased with higher NaCl concentration compared with the control plants. Therefore, the SW treatment of 102 mM NaCl resulted in the lowest protein content (0.13 mg g^−1^ DW) and essential oil yield (0.14%) compared with the control plants (Table 3). The abovementioned leaf osmoprotectant compounds increased significantly with the application of a 25 μM FA foliar spray. They further increased with 50 μM FA application compared with the control (Table 3). FA foliar treatment at 25 and 50 μM increased TSS content by 27.8 and 61.1%, protein content by 29.4 and 70.6%, free amino acids by 35.7 and 85.7%, proline by 20.0 and 44.0%, total phenolics by 30.4 and 47.8%, and essential oil yield by 27.7 and 55.6%, respectively, compared with the control. The effect of treatment with SW and FA was significant for the leaf osmoprotectant compounds of *P. amboinicus* (Table 3).

**Table 3:** The effect of SW irrigation and FA spraying on leaf osmoprotectant compounds and essential oil yield of *Plectranthus amboinicus* plants grown under greenhouse conditions.

**Treatment**	**TSS**	**Proteins**	**Amino acids**	**Proline**	**Phenolic**	**Essential oil yield (%)**
	**mg g**^**−1**^ **DW**					
SW (mM)	^*^	^*^	^*^	^*^	^*^	^*^
0 (Control)	0.21 ± 0.02c	0.32 ± 0.03a	0.17 ± 0.02c	0.28 ± 0.02c	0.26 ± 0.01c	0.32 ± 0.02a
34	0.26 ± 0.03b	0.26 ± 0.03b	0.22 ± 0.02b	0.34 ± 0.00b	0.31 ± 0.02b	0.27 ± 0.02b
68	0.31 ± 0.04a	0.19 ± 0.02c	0.29 ± 0.03a	0.40 ± 0.03a	0.39 ± 0.02a	0.19 ± 0.01c
102	0.16 ± 0.01d	0.13 ± 0.01d	0.11 ± 0.01d	0.19 ± 0.01d	0.20 ± 0.01d	0.14 ± 0.01d
FA (μM)	^*^	^*^	^*^	^*^	^*^	^*^
0 (Control)	0.18 ± 0.02c	0.17 ± 0.02c	0.14 ± 0.01c	0.25 ± 0.02c	0.23 ± 0.01c	0.18 ± 0.01c
25	0.23 ± 0.02b	0.22 ± 0.01b	0.19 ± 0.02b	0.30 ± 0.02b	0.30 ± 0.03b	0.23 ± 0.01b
50	0.29 ± 0.01a	0.29 ± 0.03a	0.26 ± 0.03a	0.36 ± 0.03a	0.34 ± 0.01a	0.28 ± 0.01a
SW × FA	^*^	^*^	^*^	^*^	^*^	^*^

* *Indicates significant differences at P ≤ 0.05 probability level*.

*According to the least-significant difference test, means ± SE followed by different letters in each column are significantly different at P ≤ 0.05*.

*SW, saline water; FA, folic acid; TSS, total soluble sugars; DW, dry weight*.

In general, the highest leaf TSS, free amino acids, proline, and phenolic content were recorded under the interactive treatment SW68 + FA50, followed by SW34 + FA 50. Conversely, the lowest TSS, free amino acids, and proline content were obtained from the interactive treatments SW102 + FA0, followed by SW0 + FA0. Concerning leaf protein content and essential oil yield, the highest mean values were recorded from the interactive treatment of SW0 + FA50, followed by SW34 + FA50, then SW68 +FA50, and finally, SW102 + FA50. However, the lowest leaf protein content and essential oil yield were recorded from the interactive treatments of SW102 + FA0 (Table 3).

### Leaf Enzymatic Activity and Some Antioxidants Content

Enzymatic and non-enzymatic systems (SOD, CAT, APX, GR, GSH, and AsA) of *P. amboinicus* increased significantly with increasing salinity levels (i.e., 34 and 68 mM NaCl). However, their levels decreased significantly under a salinity level of 102 mM NaCl compared with the control (tap water) (Table 4). SW treatments of 34 and 68 mM NaCl increased SOD by 20 and 39.2%, CAT by 9.6 and 30.3%, APX by 15.3 and 31.2%, GR by 21.9 and 42.2%, GSH by 16.7 and 40.5%, and AsA by 45.9 and 73.8%, respectively. Notably, the lowest levels of enzymatic and non-enzymatic antioxidants were obtained when the salinity level reached 102 mM NaCl compared with the control. As for FA foliar treatments, treatment of 25 or 50 μM markedly enhanced the content of enzymatic and non-enzymatic components of *P. amboinicus* compared with the control (Table 4).

**Table 4:** The effect of SW irrigation and FA spraying on leaf enzymatic activity and some antioxidant contents of *Plectranthus amboinicus* plants grown under greenhouse conditions.

**Treatment**	**SOD**	**CAT**	**APX**	**GR**	**GSH**	**AsA**
	**μmol g**^**−1**^ **FW**					
SW (mM)	^*^	^*^	^*^	^*^	^*^	^*^
0 (Control)	0.125 ± 0.01c	0.178 ± 0.01c	0.157 ± 0.01c	0.128 ± 0.01c	0.227 ± 0.02c	0.183 ± 0.02c
34	0.150 ± 0.01b	0.195 ± 0.02b	0.181 ± 0.01b	0.156 ± 0.02b	0.265 ± 0.01b	0.267 ± 0.01b
68	0.174 ± 0.02a	0.232 ± 0.4a	0.206 ± 0.02a	0.182 ± 0.02a	0.319 ± 0.01a	0.318 ± 0.02a
102	0.102 ± 0.1d	0.144 ± 0.2d	0.139 ± 0.03d	0.109 ± 0.03d	0.166 ± 0.03d	0.140 ± 0.01d
FA (μM)	^*^	^*^	^*^	^*^	^*^	^*^
0 (Control)	0.120 ± 0.01c	0.167 ± 0.02c	0.150 ± 0.03c	0.124 ± 0.01c	0.205 ± 0.01c	0.185 ± 0.01c
25	0.138 ± 0.01b	0.188 ± 0.02b	0.171 ± 0.1b	0.145 ± 0.01b	0.242 ± 0.02b	0.224 ± 0.03b
50	0.156 ± 0.01a	0.206 ± 0.01a	0.192 ± 0.1a	0.163 ± 0.02a	0.286 ± 0.01a	0.272 ± 0.03a
SW × FA	^*^	^*^	^*^	^*^	^*^	^*^

* *Indicates significant differences at P ≤ 0.05 probability level*.

*According to the least-significant difference test, means ± SE followed by different letters in each column are significantly different at P ≤ 0.05*.

*SW, saline water; FA, folic acid; SOD, superoxide dismutase; CAT, catalase; APX, ascorbate peroxidase; GR, glutathione reductase; GSH, glutathione; AsA, ascorbic acid; FW, fresh weight*.

**Figure 2:**
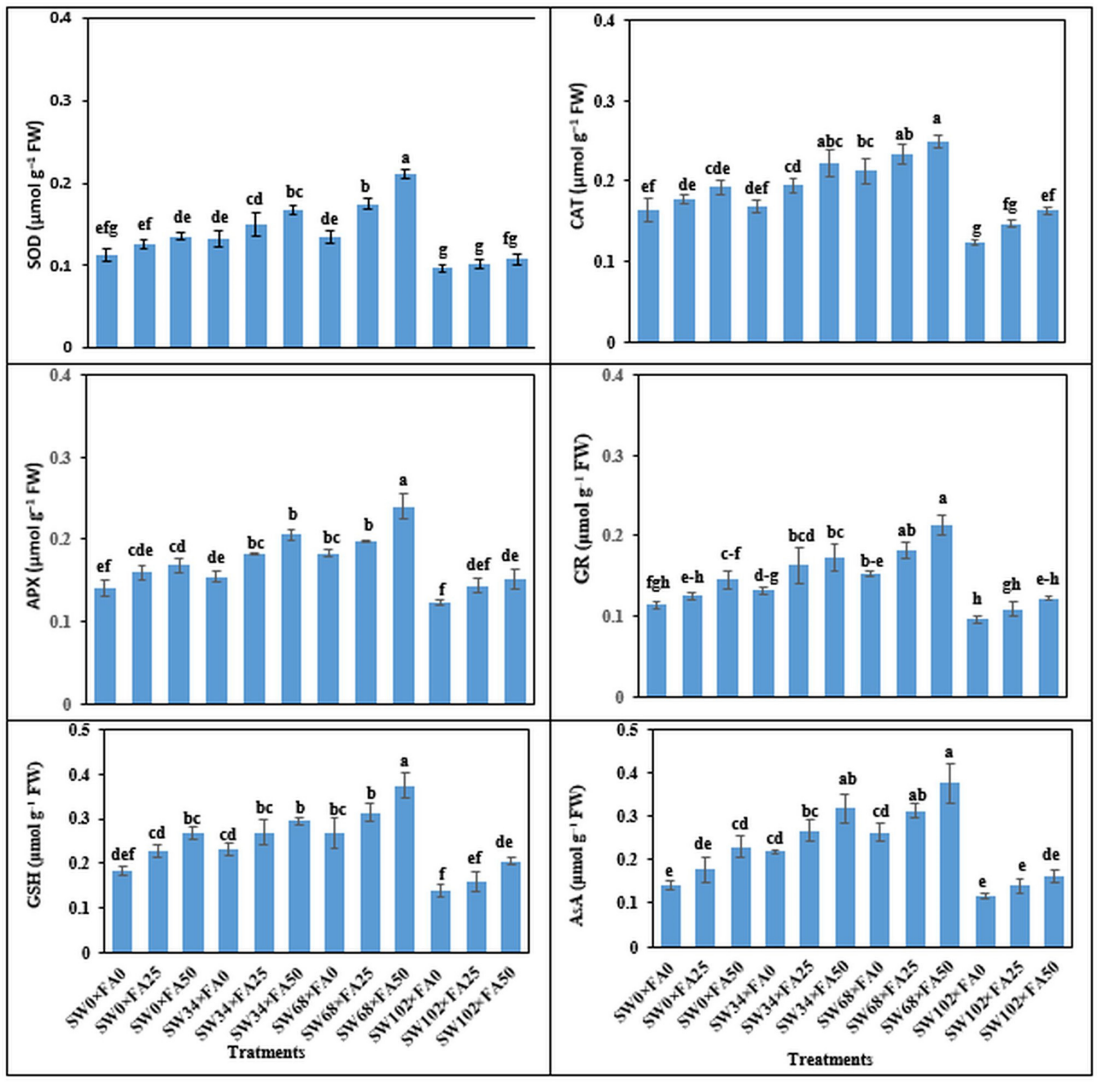
The interactive effect of folic acid (FA) and saline water (SW) on enzymatic antioxidant activity of *Plectranthus amboinicus*. **(A)** SOD; **(B)** CAT; **(C)** APX; **(D)** GR; **(E)** GSH; and **(F)** AsA activity of *P. amboinicus* the two seasons of 2018/2019 and 2019/2020. Different letters on the bars refer to significant differences among means based on Fisher's least-significant difference test at *p* < 0.05. SOD, superoxide dismutase; CAT, catalase; APX, ascorbate peroxidase; GR, glutathione reductase; GSH, glutathione; AsA, ascorbic acid; SW0, 0 mM NaCl; SW34, 34 mM NaCl; SW68, 68 mM NaCl; SW102, 102 mM NaCl; FA0, 0 μM FA; FA 25, 25 μM FA; FA50, 50 μM FA.

FA applied in foliar sprays at 25 and 50 μM increased SOD by 15.0 and 30.0%, CAT by 12.6 and 23.4%, APX by 14.0 and 28.0%, GR by 16.9 and 31.5%, GSH by 18.0 and 39.5%, and AsA by 35.7 and 47.0%, respectively, compared with the control. Treatment with SW and FA significantly affected the enzymatic and non-enzymatic antioxidants in *P. amboinicus* (Figure 2). Sequential FA application at any rate (i.e., 25 or 50 μM) under different salinity levels significantly increased enzymatic and non-enzymatic systems in comparison with untreated plants (control). In this regard, the most noticeable increments of the abovementioned antioxidants were observed with a salinity level of 34 mM NaCl × FA at 50 μM, and a further salinity level of 68 mM NaCl × FA at 50 μM.

### Mineral Content of Leaves

The salinity levels of 34, 68, and 102 mM NaCl markedly decreased the elemental leaf contents (i.e., N, P, K^+^, and the leaf K^+^/Na^+^ ratio), but positively increased the leaf contents of Na^+^ and Cl^−^, compared with the control (tap water) (Table 5). SW applied at a concentration of 102 mM NaCl decreased leaf content of N by 55.3%, P by 48.1%, K^+^ by 39.7%, and the leaf K^+^/Na^+^ ratio by 61.8%, whereas the leaf Na^+^ and Cl^−^ content increased by 52.9 and 89.6%, respectively, compared with the SW0 treatment. FA foliar treatments resulted in significant increases in the contents of leaf N, P, K^+^, and the leaf K^+^/Na^+^ ratio. However, the leaf Na^+^ and Cl^−^ content significantly decreased with foliar spray of 25 or 50 μM FA compared with the control treatment (SW0) (Table 5). FA foliar spray applied at 25 and 50 μM increased the content of N by 30.7 and 72.3%, P by 17.6 and 41.2%, K^+^ by 13.9 and 29.2%, and increased the leaf K^+^/Na^+^ ratio by 50.2 and 96.4%, respectively. The leaf Na^+^ content decreased by 12.0 and 28.0%, and Cl^−^ content decreased by 16.7 and 32.8%, respectively, compared with the control. The interaction effect between SW treatments and FA applications was significant for leaf macro- and micro-nutrient content of *P. amboinicus* (Table 5). Generally, the highest leaf N, P, K^+^ contents, and leaf K^+^/Na^+^ ratio, or the lowest leaf Na^+^ and Cl^−^ content, were obtained from the interactive treatment of non-saline water 0 mM NaCl × FA at 50 μM, followed by SW 34 mM NaCl × FA at 50 μM, then SW 68 mM NaCl × FA at 50 μM, and finally, the lowest was obtained for the treatment of SW 102 mM NaCl × FA at 50 μM. The lowest leaf contents of N, P, and K^+^ and lowest leaf K^+^/Na^+^ ratio, or the highest leaf Na^+^ and Cl^−^ content, were obtained from the interactive treatments of SW 102 mM NaCl × FA at 0 mM.

**Table 5:** The effect of SW irrigation and FA spraying on leaf elemental status of *Plectranthus amboinicus* plants grown under greenhouse conditions.

**Treatment**	**N%**	**P**	**K^**+**^**	**Na^**+**^**	**Cl^**−**^**	**K^**+**^/Na^**+**^**
		**mg g**^**−1**^ **DW**				
SW (mM)	^*^	^*^	^*^	^*^	^*^	^*^
0 (Control)	25.3 ± 0.71a	0.27 ± 0.02a	41.6 ± 1.5a	0.17 ± 0.01d	1.15 ± 0.1d	55.5 ± 2.1a
34	20.6 ± 0.81b	0.22 ± 0.02b	36.6 ± 1.5b	0.20 ± 0.02c	1.47 ± 0.1c	41.9 ± 2.4b
68	16.4 ± 0.65c	0.18 ± 0.01c	31.6 ± 1.5c	0.23 ± 0.02b	1.81 ± 0.2b	32.0 ± 2.1c
102	11.3 ± 0.48d	0.14 ± 0.01d	25.1 ± 1.3d	0.26 ± 0.02a	2.18 ± 0.2a	21.2 ± 2.0d
FA (μM)	^*^	^*^	^*^	^*^	^*^	^*^
0 (Control)	13.7 ± 0.56c	0.17 ± 0.01c	29.5 ± 1.6c	0.25 ± 0.01a	1.98 ± 0.2a	25.3 ± 1.8c
25	17.9 ± 0.78b	0.20 ± 0.01b	33.6 ± 1.3b	0.22 ± 0.02b	1.65 ± 0.2b	38.0 ± 2.6b
50	23.6 ± 0.65a	0.24 ± 0.02a	38.1 ± 1.4a	0.18 ± 0.02c	1.33 ± 0.2c	49.7 ± 2.0a
SW × FA	^*^	^*^	^*^	^*^	^*^	^*^

* *Indicates significant differences at P ≤ 0.05 probability level*.

*According to the least-significant difference test, means ± SE followed by different letters in each column are significantly different at P ≤ 0.05*.

*SW, saline water; FA, folic acid; DW, dry weight*.

### Anatomical Stem Structure

**Figure 3:**
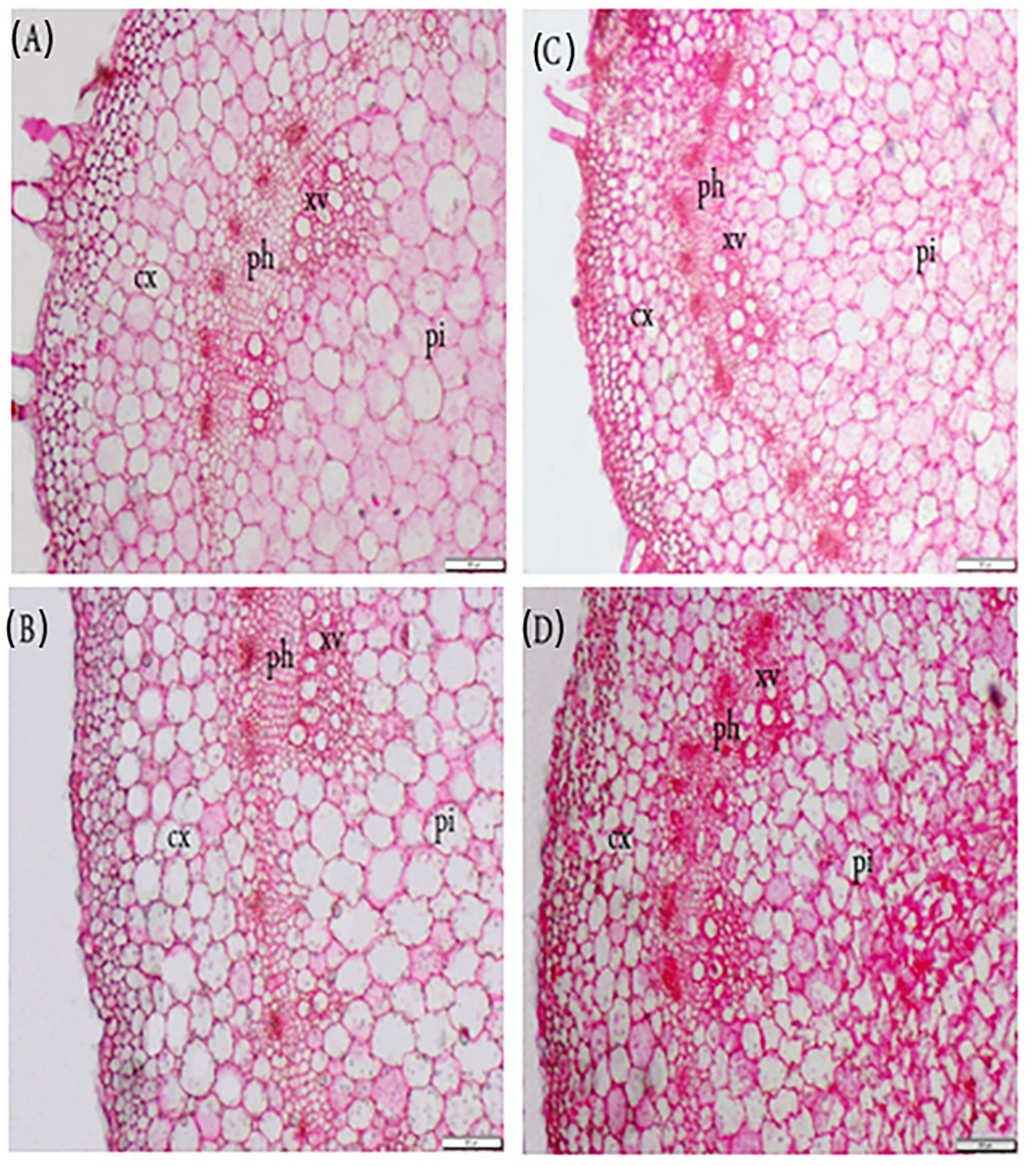
Exogenous application of folic acid (FA) and saline water (SW) on the stem anatomical structure of *Plectranthus amboinicus*. **(A)** FA_0_ SW_0_; **(B)** FA_50_ SW_0_; **(C)** FA_0_ SW_102_; and **(D)** FA_25_SW_102_. Cx, cortex; ph, phloem; xv, xylem vessels; pi, pith; SW0, 0 mM NaCl; SW102, 102 mM NaCl; FA0, 0 μM FA; FA 25, 25 μM FA; FA50, 50 μM FA.

The impact of FA on the morphological characteristics of *P. amboinicus* watered with saline water was also assessed (Table 6, Figure 3). At all levels of salt stress, the application of FA had a stimulating effect on stem structure. All anatomical attributes dropped dramatically with increased salinity irrigation level. Overall, in the absence of salinity stress, FA application resulted in a significant increase in stem diameter, especially at high doses. However, exogenous FA alleviated the inhibitory effect of salt stress. The highest stem diameter was observed in plants treated with distilled water and 50 μM FA (an increase of 22.27% compared with control). This increase in stem diameter led to increases in cortex thickness, pith thickness, phloem tissue thickness, xylem tissue thickness, and xylem vessel diameter by 14.29, 34.5, 19.35, 15.0, and 30.0%, respectively, compared to control plants. It is noteworthy that the decrease in stem diameter due to the increase in SW irrigation from 34 to 102 mM NaCl was compensated by FA application, and a significant stem diameter decrease was recorded for the high level of salt stress. Application of FA 50 μM resulted in stem diameter increases by 10.18, 9.66, and 119.25%, in plants irrigated with SW at concentrations 102, 68, 34, and 0 mM, respectively, and resulted from increases in cortex thickness by 0, 26.77, and 33.33 %, respectively. FA spray of 50 μM in plants irrigated with SW at concentrations 34, 68, and 102 mM resulted in increases in phloem thickness by 12.5, 11.11, and 26.32%, xylem thickness by 10, 6.67, and 6.9%, xylem vessel diameter by 10, 12.5, and 10%, pith thickness by 9.14, 16.15, and 14%, respectively, compared with the control.

**Table 6:** The effect of SW irrigation and FA spraying on the anatomical stem structures of *Plectranthus amboinicus* plants grown under greenhouse conditions.

**Treatment**	**Stem diameter**	**Cortex thickness**	**Phloem thickness**	**Xylem thickness**	**Vessel diameter**	**Pith thickness**
	**(μm)**	**(μm)**	**(μm)**	**(μm)**	**(μm)**	**(μm)**
SW (mM)	^*^	^*^	^*^	^*^	^*^	^*^
0 (Control)	4312.0 ± 7.0a	375.0 ± 2.7a	186.7 ± 2.2a	230.0 ± 2.6a	58.3 ± 1.6a	2762.0 ± 5.8a
34	4008.0 ± 6.3b	362.3 ± 3.4b	170.0 ± 2.5b	226.7 ± 2.6b	51.8 ± 1.5b	2399.7 ± 4.2b
68	3845.7 ± 6.5c	320.3 ± 2.6c	135.0 ± 2.4c	160.0 ± 2.4c	47.1 ± 1.4c	2249.7 ± 3.9c
102	2487.3 ± 5.8d	266.7 ± 3.1d	111.7 ± 2.2d	160.0 ± 2.2c	44.0 ± 1.6d	2170.7 ± 3.3d
FA (μM)	^*^	^*^	^*^	^*^	^*^	^*^
0 (Control)	3278.0 ± 6.8c	303.0 ± 2.9c	136.3 ± 2.2c	173.8 ± 2.6c	45.5 ± 1.4c	2159.0 ± 4.3c
25	3509.0 ± 6.4b	334.3 ± 3.0b	157.5 ± 2.2b	191.3 ± 2.4b	50.7 ± 1.6b	2456.0 ± 4.8b
50	4203.8 ± 6.1a	356.0 ± 3.0a	158.8 ± 2.5a	217.5 ± 2.3a	54.8 ± 1.5a	2571.5 ± 3.8a
SW × FA	^*^	^*^	^*^	^*^	^*^	^*^

* *Indicates significant differences at P ≤ 0.05 probability level*.

*According to the least-significant difference test, means ± SE followed by different letters in each column are significantly different at P ≤ 0.05*.

*SW, saline water; FA, folic acid*.

## Discussion

Plant morphological and physiochemical attributes are significantly affected by salinity. One of the earliest reactions of crops to salinity is the reduction in leaf development rate (Franco et al., 1997); the excess salt surrounding the roots causes osmotic pressure, which reduces the supply of water to leaf cells. Root growth can be inhibited by high external salt absorption (Krzymińska and Ulczycka-Walorska, 2015), resulting in reduced root length (Shannon and Grieve, 1998) and function. Cell elongation and leaf division are reduced, causing a reduction in leaf area (Munns and Tester, 2008).

A decrease in turgor in the leaves as a result of changes in cell wall characteristics or a fall in photosynthetic rate could induce a decrease in leaf area (Franco et al., 1997). The remarkable reduction of morphological characteristics of *P. amboinicus* are attributed to different levels of salt in irrigation water. Such consequences have been observed in several plant species including *Plectranthus forsteri* (Krzymińska and Ulczycka-Walorska, 2015), *P. amboinicus* (Karimian et al., 2019), and lavender (*Salvia splendens*) (Paraskevopoulou et al., 2020). On the other hand, significant increases in the abovementioned growth parameters of *P. amboinicus* compared with the control (spray with water) were observed when FA was applied as a foliar spray at concentrations of 25 or 50 μM.

One of the most important cellular functions of FA is that it serves as a co-factor for crucial enzymes for RNA and DNA synthesis, and has a stimulating effect on plant growth and development (Scott et al., 2000). In addition, FA deficiency slows down nucleic acid synthesis and interrupts cell division (Kim et al., 2009). FA also plays a critical role in maintaining genome stability and protecting the metabolism and DNA of plants (Fenech, 2001). It has been long known that FA increases mitotic division, thereby increasing plant growth and development (Hillis et al., 2011). Emam et al. (2011) showed that foliar application of vitamins including FA (B9) markedly increased the growth parameters of flax (*Linum usitatissimum*) plants compared with the control. A similar trend was reported by Farouk and El-Saidy (2013) and Heo et al. (2019) on sunflower and snapdragons (*Antirrhinum majus*) plants, respectively. Folic acid-treated plants had a stronger stimulatory impact, possibly because of its effect on protein and nucleic acid biosynthesis regulation (Andrew et al., 2000).

Salt stress suppresses photosynthesis by reducing leaf photosynthetic pigments (SPAD index), chlorophyll fluorescence, and its performance (i.e., Fv/Fm and PI). Furthermore, salt stress causes stomatal closure, which reduces CO_2_ availability in the leaves and prevents carbon fixation. Salt stress also exposes chloroplasts to high excitation energy, which increases the production of ROS like O_2_•-, H_2_O_2_, OH•, and singlet oxygen atoms (1O_2_) (Parvaiz and Satyawati, 2008; Abd El-Mageed et al., 2021b). Accumulating dangerous ions (Na^+^ and/or Cl^−^) under salt stress conditions may hinder photosynthesis and protein production, deactivate enzymes and destroy chloroplasts and other organelles (Taiz and Zeiger, 2002). The reduction in chlorophyll capacity and photosynthetic rates under salt stress are described in several studies (Semida et al., 2014, 2015a).

In the present study, a positive increase was observed in the phytochemical constituents of *P. amboinicus* with a FA foliar spray compared with the control plants. This observation was supported by the study demonstrating a reduction in whole folate abundance and chlorophyll level in pea leaves upon application of the anti-folate drug MTX (Van Wilder et al., 2009). Also, Stakhova et al. (2000) found that FA had a favorable effect on plant growth. Pretreating barley plants with FA significantly boosted their photosynthetic apparatus and reduced the negative effects of salt stress (Kiliç and Aca, 2016). The increase of TSS, amino acids, proline, total phenolic content, and decrease in crude protein content observed in *P. amboinicus* with increasing salinity levels proves this plant's tolerance to salt stress. The sugars act as respiratory substrates or as osmolytes to confer salinity stress tolerance (Kotagiri and Kolluru, 2017).

Osmoprotectants are neutral compounds that protect proteins and membranes from salt denaturation (Munns, 2002). Furthermore, plant cells modify their osmotic potential to avoid water loss and preserve cell turgor when exposed to salinity (Naidoo and Naidoo, 2001). The increased content of soluble sugars and proline under salinity environments was described by Ramezani et al. (2011). Proline accumulation may occur either due to protein degradation or inhibition of proline conversion under salinity (Singh et al., 1973). Proline has also been recommended as a ROS scavenger and molecular chaperone (Verbruggen and Hermans, 2008). Its accumulation can improve the structure of membranes and proteins to reduce cell destruction under salinity. It has been proved that increasing amino acid content may be due to free-radical scavenging, osmotic adjustment, and protein and membrane integrity (Keutgen and Pawelzik, 2008). These findings are consistent with those of Semida et al. (2019).

Osmoprotectants are substances that can intercept and neutralize the harmful effects of oxygen-free radicals generated during normal cellular metabolism (Benzie, 2003). As seen in Table 3, osmotic adjustment substances (i.e., TSS, proteins, amino acids, proline, and phenolics) increased significantly with foliar application of FA treatments compared with the control. Application of vitamins like FA enhances alpha-ketoglutaric acid biosynthesis, which combines with NH_3_ to form amino acids and proteins, thereby promoting the creation of natural hormones such as IAA, cytokinins, and gibberellins. These, in turn, encourage cell division, accumulation of pigments and enzymes, and plant metabolism (Samiullah et al., 1988). The increase in vegetative growth characteristics caused by FA may be attributed to enhancing the accumulation of leaf osmoprotectants. For example, phenolics play a significant role in regulating plant metabolic processes and overall plant growth, as well as lignin synthesis (Lewis and Yamamoto, 1990). Enhancement of plant growth responses by FA under salt stress is closely linked to phenolic stimulation that occurred via the pentose phosphate pathway. Such stimulation resulted in overexpression of phenolic contents and GPX activity for the structural development and extra phenolics in late free-radical scavenging-linked antioxidant activity (Burguieres et al., 2007).

Additionally, the application of FA increased proline content under salinity stress, developing plant resistance against stress (Burguieres et al., 2007). Our results are consistent with those obtained by El-Metwally and Dawood (2017), who found that the percentages of TSS and phenolic compounds in fava bean were appreciably influenced by FA. Ionic effects of salinity bring accumulation or reduction of specific secondary metabolites (Mahajan and Tuteja, 2005) such as phenols, which are known to increase under stressful conditions and help plants bring osmotic balance. In this study, there was an increase in phenolic content by 30.0, 55.0, and 95.0% at salinity levels of 34, 68, and 102 mM NaCl, respectively, compared with the control. Hussein and Alva (2014) indicated that the concentration of total phenols in pepper (*Capsicum annuum* L.) leaves significantly increased with an increase in irrigation water salinity. These results are consistent with those obtained by Ali et al. (2013). The decrease in growth was linked to a lower osmotic potential in the soil, which resulted in decreased water intake, reduced transpiration, and stomatal closure (Ben-Asher et al., 2006). A similar study by Abdelrazik et al. (2016) reported that the essential oil yield significantly decreased when irrigation water salinity was increased to the highest level.

Salinity causes oxidative stress in plants (Rout and Shaw, 2001). ROS, created during metabolism, are the source of oxidative damage (Hasanuzzaman et al., 2020). ROS can harm membrane lipids, proteins, and nucleic acids (Noctor and Foyer, 1998). To minimize the effect of oxidative stress caused by salinity, a plant cell has developed an antioxidant system that includes compounds such as GSH and AsA, in addition to antioxidant enzymes such as SOD, CAT, APX, and GR, which scavenges ROS (Apel and Hirt, 2004). The increase of antioxidant enzyme activity as a response to salinity stress could indicate increasing ROS and the development of a defensive mechanism to mitigate the oxidative damage caused by salt stress (Chaparzadeh et al., 2004). Our results indicated a significant increase in enzymatic and non-enzymatic antioxidants (SOD, CAT, APX, GR, GSH, and AsA) of *P. amboinicus* leaves with increasing salinity levels up to 68 mM NaCl. The levels subsequently declined significantly when exposed to the highest salinity level of 102 mM NaCl compared with the control plants (tap water). The high levels of SOD are necessary to convert the superoxide radical ${\text{O}}_{2}^{-}$ to H_2_O_2_, which is scavenged by CAT, APX, and GPX, protecting plants from the negative effects of ROS (Badawi et al., 2004). Elevated SOD activity without scavenging H_2_O_2_, can result in cytotoxicity by virulent OH• generated from H_2_O_2_ (Gossett et al., 1994).

Furthermore, plants sprayed with FA showed a significant improvement in the scavenging activity of H_2_O_2_, as evidenced by a steady rise in CAT and APX activities up to moderate salinity levels, resulting in decreased H_2_O_2_ content. Currently, foliar-feeding with FA in *P. amboinicus* can ameliorate the oxidative stress induced by salinity; moreover FA application at 50 μM markedly enhanced levels of enzymatic and non-enzymatic antioxidants under SW irrigation levels of 34 or 68 mM NaCl. Marked increases in ascorbate and glutathione were observed in salinized *P. amboinicus* plants treated with FA. Glutathione plays a significant role in the defense against oxidative stress. The AsA/GSH cycle is complex and adapts the protein thiol-disulfide redox status of plants in response to abiotic and biotic stresses (Mullineaux and Rausch, 2005). Decreases in ascorbate and glutathione in response to salinity were observed by Emam et al. (2011). Furthermore, the enhancement in SOD and GR activities was followed by increased levels of polyphenols and ascorbate content. Increasing salinity in irrigation water from 34 to 102 mM NaCl caused significant increases in Na^+^ and Cl^−^ accumulation in the dry leaves of *P. amboinicus*. Meanwhile, the levels of N, P, K^+^, and the K^+^/Na^+^ ratio significantly decreased compared with control plants.

Salinity severely affected crop performance, especially nutritional disorders, in which the relationships between salinity and mineral nutrition in crops are complex (Maas and Grattan, 1999). The increase of salts in the cell apoplast causes ionic toxicity, imbalance, and hyperosmotic activities because the increase of Na^+^ and Cl^−^ inhibits the processes of cytosol and organelles (Zhu et al., 1998).

Several studies indicated that salinity reduces the absorption and accumulation of nutrients in the plant (Hu and Schmidhalter, 2005). Nitrogen absorption is reduced due to the competition between sodium and ammonium or chloride with nitrates, which reduces the growth and productivity of crops (Rozeff, 1995). In addition, nitrate absorption decreases due to chloride competition or low water absorption under saline conditions (Lea-Cox and Syvertsen, 1993; Bar et al., 1997). Furthermore, phosphorous availability decreases in saline soils because salts reduce phosphate activity. Since sodium competes with potassium for absorption sites in the roots or transport in the xylem, increasing sodium in the soil reduces the absorption of potassium and calcium (Abd El-Mageed et al., 2021b; Qadir et al., 2021). Additionally, an increase in sodium chloride leads to potassium disturbance, which causes defects in cell membranes, photosynthesis, and other vital processes, the generation of ROS, and cell death (Chakraborty et al., 2016).

Potassium is a vital plant nutrient, but sodium competes with it for absorption in cells, especially when its concentration is higher than potassium (Rodriguez-Navarro, 2000). These results are consistent with the results from Abd El-Mageed et al. (2021a). Our results reflect the functional role of FA as a foliar spray in enhancing nutrient uptake; FA application yielded the highest values of N, P, and K^+^ as well as leaf K^+^/Na^+^ ratio, in contrast to decreased leaf Na^+^ and Cl^−^ contents when compared with the control (sprayed with water). The positive effects of B-vitamins on the growth and development of *P. amboinicus* might be due to their crucial role in protecting plant cells from aging and several disorders, as well as enhancing cell division, natural hormone biosynthesis, and uptake of nutrients and water (Samiullah et al., 1988). Emam et al. (2011) clarified that under salinity stress, Na^+^ noticeably accumulated in salinized flax seedlings, while K^+^ concentration and the K^+^/Na^+^ ratio significantly decreased as salinity levels increased.

FA increases growth traits through increased absorption of essential elements by binding to them, which increases cell division and differentiation (Poudineh et al., 2015). All anatomical attributes decreased with increased levels of salinity irrigation; these decreases could be attributed to the negative effect of salt stress to decrease the measured characteristics (i.e., stem diameter, thicknesses of the cortex, phloem, and xylem; xylem vessel diameter, and pith thickness) (Abd El-Mageed et al., 2018). In contrast, FA application improved all anatomical attributes; this amelioration resulted from increases in the thicknesses of plant cortex, phloem, xylem, and xylem vessel diameter and pith thickness. These increases may be due to the regulatory role that FA may have in DNA function, thereby increasing cell division (Kiliç and Aca, 2016). All improved characteristics resulting from foliar application of FA were accompanied by an ameliorated stem anatomy (Table 6), giving the plants a chance for sufficiently healthy cell metabolic processes. FA foliar application has a positive effect on *P. amboinicus* growth and yield under conditions of saline water irrigation.

## Conclusion

The results of this study clearly showed that the application of FA reduces the inhibitory effects of saline water stress on *P. amboinicus*. In addition, FA can boost the antioxidant activity (AsA, SOD, GSH, GR, APX, and CAT) and osmolytes accumulation (proline, free amino acids, TSS, K^+^/Na^+^, and K^+^) as well as increase the phenolic and protein contents. Application of FA increases SPAD, Fv/Fm, PI, nutrient acquisition, and oil yield, consequently promoting growth and productivity of salt-stressed *P. amboinicus*. Thus, future applications of the biostimulant, FA, can be used to improve plant performance under salt stress conditions.

## Data Availability Statement

The original contributions presented in the study are included in the article/supplementary material, further inquiries can be directed to the corresponding authors.

## Author Contributions

OA-E, TAE-M, ME-S, SA, and KE-T conceived and designed the research. OA-E, KH, MA-R, TAE-M, ME-S, SA, KE-T, and RT supervised the study. OA-E, KH, MA-R, TAE-M, ME-S, and RT performed greenhouse experiments. OA-E, KH, TAE-M, and RT performed the microscopic experiments. OA-E, MA-R, TAE-M, ME-S, SA, KE-T, and RT analyzed the data. SA and KE-T assisted with experiments and/or data evaluation. TAE-M, ME-S, SA, KE-T, and RT wrote the manuscript. All authors critically revised the manuscript and approved the final version.

## Funding

This project was funded by the Khalifa Center for Biotechnology and Genetic Engineering-UAEU (Grant #: 31R286) to SA and the Abu Dhabi Award for Research Excellence-Department of Education and Knowledge (Grant #: 21S105) to KE-T.

## Conflict of Interest

The authors declare that the research was conducted in the absence of any commercial or financial relationships that could be construed as a potential conflict of interest.

## Publisher's Note

All claims expressed in this article are solely those of the authors and do not necessarily represent those of their affiliated organizations, or those of the publisher, the editors and the reviewers. Any product that may be evaluated in this article, or claim that may be made by its manufacturer, is not guaranteed or endorsed by the publisher.
